# Long- and short-term outcomes of balloon dilation for benign choledochojejunal anastomotic stricture using balloon endoscopy-assisted ERCP: a multi-center retrospective cohort study

**DOI:** 10.1186/s12876-023-02830-3

**Published:** 2023-06-01

**Authors:** Yuhei Iwasa, Takuji Iwashita, Keisuke Iwata, Shinya Uemura, Mitsuru Okuno, Ryuichi Tezuka, Akihiko Senju, Tsuyoshi Mukai, Masahito Shimizu

**Affiliations:** 1 Department of Gastroenterology, Gifu Municipal Hospital, 7-1 Kashima-Cho, Gifu City, Gifu 500-8513 Japan; 2 First Department of Internal Medicine, Gifu University Hospital, 1-1 Yanagido, Gifu City, Gifu 501-1194 Japan; 3 Department of Gastroenterological Endoscopy, Kanazawa Medical University, Ishikawa, Japan

**Keywords:** Choledochojejunal anastomotic stricture, Pancreaticoduodenectomy, Balloon endoscopy-assisted ERCP, Balloon dilation

## Abstract

**Background:**

Benign choledochojejunal anastomotic stricture (CJS) is a common complication of pancreaticoduodenectomy and choledochojejunostomy. CJS is generally treated with balloon dilation, using balloon endoscopy-assisted endoscopic retrograde cholangiopancreatography (BE-ERCP); however, its long- and short-term outcomes have not been fully evaluated. Therefore, we evaluated the treatment outcomes of balloon dilation with BE-ERCP for CJS.

**Methods:**

We retrospectively analyzed 40 patients who had undergone balloon dilation with BE-ERCP for CJS between January 2009 and December 2022. The primary outcomes were technical and clinical success, and adverse event rates of balloon dilation using BE-ERCP for CJS. The secondary outcomes were long-term treatment outcomes for CJS recurrence, and evaluation of risk factors for recurrence.

**Result:**

Technical and clinical success rates were 93% (37/40) and 100% (37/37), respectively. CJS recurrence occurred in 32% (20/37). No procedure-related adverse events were observed. The significant risk factors of CJS after balloon dilation were its early occurrence after surgery (unit hazard ratio [HR] for month, 0.87; 95% confidence interval [CI], 0.76–0.99; *p*-value = 0.04) and residual waist during balloon dilation (HR, 5.46; 95% CI, 1.18–25.1; *p*-value = 0.03). Receiver operating characteristic curve analysis of time from surgery to balloon dilation revealed an area under the curve of 0.80 (95% CI, 0.65–0.94) and the cut-off value was 13.2 months.

**Conclusion:**

Treatment of CJS with balloon dilation was effective, although CJS recurrence occurred in one-third of the patients. The risk factors for recurrence were early occurrence of CJS after surgery and remaining waist circumference during balloon dilation.

## Background

Choledochojejunal anastomotic stricture (CJS) is a major late adverse effect after hepatectomy, biliary reconstruction, or pancreaticoduodenectomy (PD), and its incidence rates are 3–12% in patients who undergo these surgeries [1, 2]. CJS can cause various symptoms, such as obstructive jaundice, cholangitis, bile duct stones, or liver abscesses, and often requires therapeutic intervention for the stricture. In the past, percutaneous transhepatic biliary drainage (PTBD) and treatments through the PTB route were mainly performed, because the surgically altered anatomy hindered endoscopic insertion into the anastomosis and the maneuverability of the endoscope. However, management through the PTB route requires percutaneous external derange, which deteriorates the quality of life and requires long-term hospitalization [3].

In 2001, Yamamoto et al*.* developed double-balloon endoscopy (DBE), which allows the deep insertion of the endoscope into the small intestine. This development i.e., the DBE, has paved the way for BE-assisted ERCP (BE-ERCP) as a new endoscopic procedure, since BE allowed to reach the biliary orifice more certainly even in surgically altered anatomy, in which a conventional endoscope could not reach due to the long distance to the biliary orifice [4]. Initially, there were limitations in the device selection and available techniques for BE-ERCP because of the longer scope length and smaller channel diameter. However, in recent years, DBE with a larger channel size and shorter scope length has been developed, expanding the number of applicable devices and techniques for ERCP. This Short-type DBE has led to improvements in the technical success rate of the procedure [5].

For the management of benign biliary strictures, mechanical dilation using a balloon catheter or bougie dilator by ERCP is the first choice recommended by the Asia–Pacific Consensus Guidelines for the endoscopic management of benign biliary strictures [6] and CJS is mainly treated by balloon dilation. Several studies have evaluated the feasibility and short-term efficacy of balloon dilation for CJS; however, the long-term outcomes have not yet been well evaluated. Therefore, this study evaluated the long-term outcomes as well as the short-term outcomes of BE-ERCP for CJS.

## Patients and methods

### Study design

This was a multicenter retrospective cohort study conducted at Gifu Municipal Hospital and Gifu University Hospital between October 2010 to October 2021. A database analysis including all BE-ERCP procedures was performed to identify patients who met the inclusion criteria. These inclusion criteria were patients who underwent BE-ERCP for benign CJS, which is defined as intrahepatic bile duct dilation (> 4 mm) on imaging studies, accompanied by elevated serum hepatobiliary enzymes (> 1.5 × upper limit), or fever-up (body temperature > 38℃). Patients who met the following criteria were excluded: 1) CJS caused by malignancy or 2) a follow-up period of less than 6 months after the initial procedure.

### Ethics approval and consent to participate

This study was conducted in accordance with the principles of the Declaration of Helsinki. The study protocol was approved by the Institutional Review Board of each institution. Informed consent was obtained from all the study participants and their legal guardians involved in this study.

### Endoscopic procedure

**Fig. 1:**
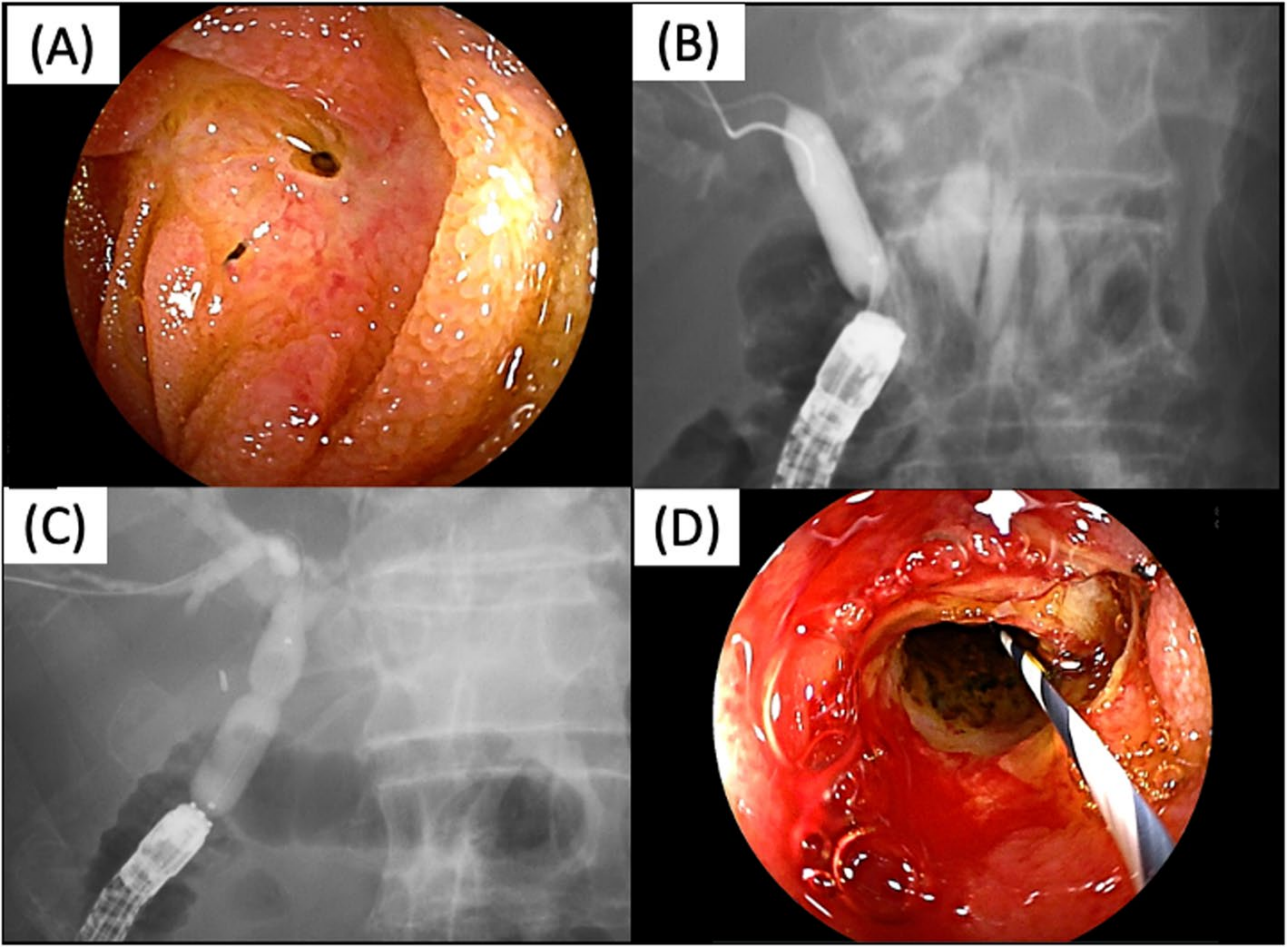
The figure of balloon dilation. **A** The anastomosis with pinhole-like choledochojejunal anastomotic stricture. **B**, **C** The patient with complete disappearance of waist during balloon dilation (**B**) and with residual waist (**C**). **D** The anastomosis after balloon dilation

A short-type DBE with a working endoscopy length and a 3.2-mm-wide working channel (EI-580BT; Fujifilm, Tokyo, Japan) was used for all procedures. The procedure was performed under moderate sedation with intravenous injections of midazolam and pentazocine. After finding the anastomosis (Fig. 1A), a wire-loaded cannulation method using a straight cannula (MTW ERCP catheter, MTW-Endoskopie Manufaktur, Wesel, Germany) and a 0.025-inch guidewire (VisiGlide 2, Olympus, Tokyo, Japan; M-Through, Asahi Intecc, Aichi, Japan; EndoSelector, Boston Scientific, Tokyo, Japan) was used for biliary cannulation. After deep biliary cannulation, a contrast agent was injected into the bile duct to evaluate biliary system configuration. Balloon dilation was performed using a balloon catheter (Hurricane RX, Boston Scientific Japan, Tokyo, Japan or REN, Kaneka Medix Corp., Osaka, Japan), with the size of 6–12 mm according to the diameter of the bile duct, just above the stricture and dilated CJS for 30–60 s (Fig. 1B, C, D).

### Study outcomes, definition and statistical analysis

The primary outcomes were technical success, clinical success, and adverse event rates of BE-ERCP for the CJS. Secondary outcomes were long-term treatment outcomes for CJS recurrence and evaluation of risk factors for recurrence.

Technical and clinical success was defined as successful balloon dilation of the CJS, and improvement in clinical symptoms within 14 days after BE-ERCP, respectively. Adverse events associated with BE-ERCP and their severities were evaluated according to the American Society for Gastrointestinal Endoscopy [7]. CJS recurrence was defined as recurrent symptoms related to CJS after initial treatment.

**Fig. 2:**
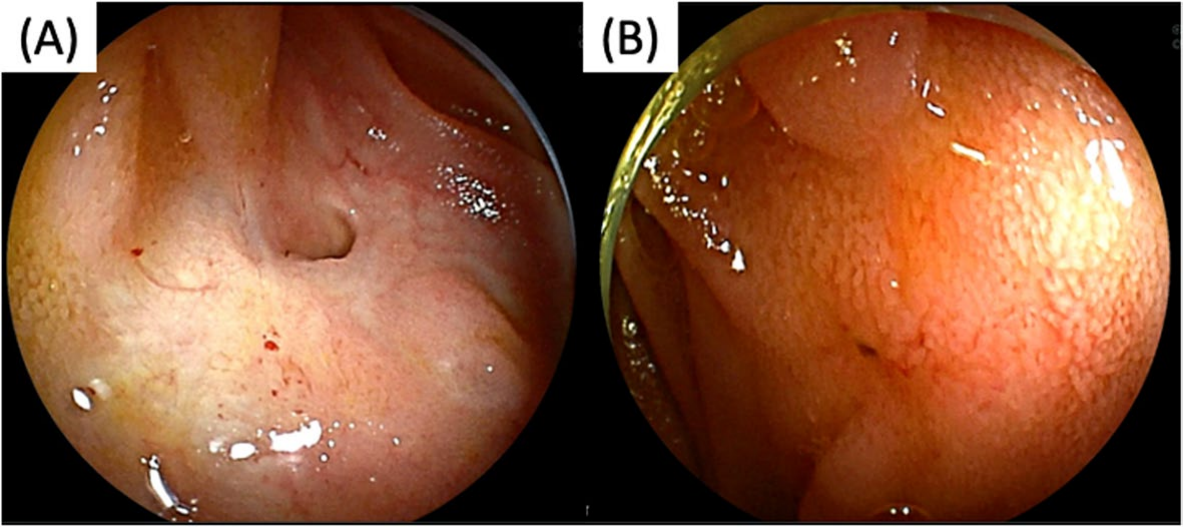
**A** The anastomosis with scar-like appearance and (**B**) without scar-like appearance

Categorical or nominal variables were compared using Fisher’s exact test, and continuous variables were compared using the Mann–Whitney U test. The time to recurrence of CJS was estimated using the Kaplan–Meier method. Continuous variables were presented as median values with minimum and maximum ranges. The possible risk factors for recurrence were evaluated using the COX proportional hazards model. All results were expressed as hazard ratios and 95% confidence intervals (CI). To identify risk factors for CJS recurrence, factors with p values < 0.05 were further analyzed in univariate analysis, using a multivariate Cox model. If the risk factors were continuous variables, receiver characteristic operating curve (ROC) analysis was performed to evaluate the area under the curve and optimal cut-off value. The following variables were analyzed: sex, age (years), type of surgical reconstruction, whether the primary disease was benign or malignant, time from surgery to balloon dilation (months), diameter of the bile duct above the CJS (mm), balloon diameter (mm), residual waist during balloon dilation, presence of bile duct stones, and presence of a scar-like appearance (obtained from previous reports [8], Fig. 2A, B). A *p*-value of < 0.05 with two-sided tests was defined as statistically significant. All statistical analyses were performed using EZR14 (version 1.61; Saitama Medical Center, Jichi, Japan).

## Results

### Basic characteristics

**Fig. 3:**
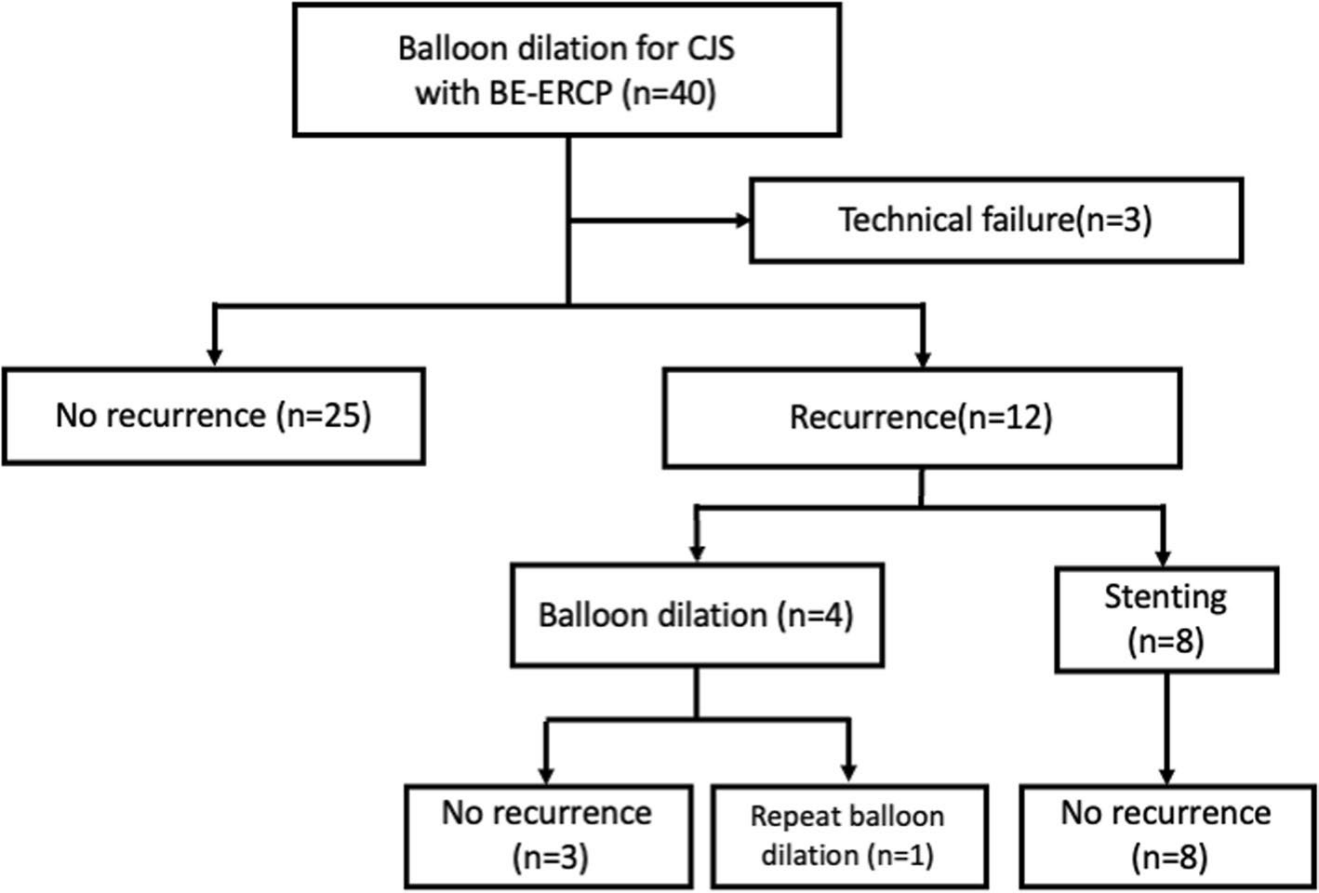
Clinical outcomes of patients who underwent balloon dilation with balloon endoscopy-assisted endoscopic retrograde cholangiopancreatography for choledochojejunostomy anastomotic stricture

A total of 40 patients (26 men; median age, 71-year-old ranging 51–83), who underwent BE-ERCP for CJS were included. Surgical reconstructions included PD with modified Child’s reconstruction, PD with other reconstructions in 4 patients, and biliary reconstruction with CJ in 30, 4, and 6, respectively. The primary diseases were pancreatic cancer in 21 patients, intraductal papillary neoplasm in 7, cholangiocarcinoma in 3, duodenal papillary cancer/adenoma in 2, neuroendocrine tumor in 2, and other diseases in 6 patients. Clinical symptoms or abnormal findings of laboratory data included elevated liver enzymes, fever, jaundice, and abdominal pain in 38, 29, 5, and 1 patients, respectively (overlapping cases). The median time from surgery to the first endoscopic treatment was 11.5 months (range, 0.8–97.5). Table 1 and Fig. 3 summarize treatment outcomes.

**Table 1:** Patient characteristics

Sex (male/female), n	26/14
Age (median, range)	71 (51–83)
Primary disease, n	
Pancreatic cancer	21
IPMN	7
Cholangiocarcinoma	3
Duodenal papillary cancer	2
Neuroendocrine tumor	2
others	5
Surgical reconstruction, n	
PD with modified Child's method	30
PD with others	4
Choledochojejunostomy with R-Y	6
The median time from surgery to first endoscopic treatment, month (range)	11.1 (0.8–97.5)
Clinical symptoms or abnormal findings of laboratory data (overlapping cases), n	
elevation of liver enzyme	38
fever	29
jaundice	5
abdominal pain	1
Technical success, n	37/40 (93%)
failure of scope insertion	2
failure of finding anastomosis site	1
Rescue treatment for technical failure, n	
PTBD rendezvous	2
conservative treatment	1
Clinical success, n	37/37 (100%)

*IPMN* Intraductal papillary mucinous neoplasm, *PD* Pancreaticoduodenectomy, *R-Y* Roux-en-Y, *PTBD* Percutaneous transhepatic biliary drainage

### Outcomes of BE-ERCP

The technical success rate was 93% (37/40), which could not be achieved in three out of 40 patients (7%), because the scope could not be inserted into the anastomosis in two patients, and the anastomosis site could not be found in one patient. Balloon dilation was performed in 37 patients, and clinical success was achieved in all patients. No adverse event-related procedures were recognized. In 3 technically unsuccessful patients, bile duct cannulation and balloon dilation using the PTBD rendezvous technique were performed as a rescue treatment in 2 patients. In the remaining 1 patient, CJS was successfully managed with conservative treatment. Table 2 lists the details of initial balloon dilation. Of the 37 patients who underwent initial balloon dilation using 6–12 mm diameter balloons, 18 had residual waist during balloon dilation. Biliary stones were found in 9 patients during the procedure, and were successfully managed after balloon dilation, using a basket catheter or balloon catheter. A scar-like appearance was observed in 12 patients. Recurrence of stricture occurred in 12 patients (32%) with a median follow-up period of 21.1 months (range, 2.0–122.6) after the initial balloon dilation. Fully covered metallic stent placement for stricture recurrence was performed in 8 of 12 patients and were removed endoscopically within 3 months without no CJS recurrence (a median follow-up period of 13.3 months range, 7.0–30.8). In the remaining four patients, repeat balloon dilation was performed, no recurrence was recognized except for one patient who required another balloon dilation in 4 months.

**Table 2:** Details of initial balloon dilation treatment

Total, n	37
Gender, n (male/female)	25/12
Age, median (range)	70 (51–83)
Primary disease, n	
Malignant disease	26
Pancreatic cancer	19
Cholangiocarcinoma	3
Duodenal papillary cancer	2
Neuroendocrine tumor	2
Benign disease	11
IPMN	7
others	4
Surgical reconstruction, n	
PD with modified Child’s method	30
PD with others	2
Choledochojejunostomy	5
The median time from surgery to first endoscopic treatment, months (range)	11.5 (0.8–97.5)
Diameter of bile duct, mm, median (range)	7 (2–13.9)
Balloon diameter, n	
6 mm/8 mm/10 mm/11 mm/12 mm	5/21/6/3/2
Residual waist during balloon dilation, n	18
Presence of biliary stone, n	21
Presence of Scar like appearance, n	12
Restenosis, n	12 (32%)
Time to CJS recurrence after balloon dilation, days	8.4 (0.5–15.4)

*IPMN* Intraductal papillary mucinous neoplasm, *PD* Pancreaticoduodenectomy

### Risk factors for recurrence of stricture after balloon dilation

**Fig. 4:**
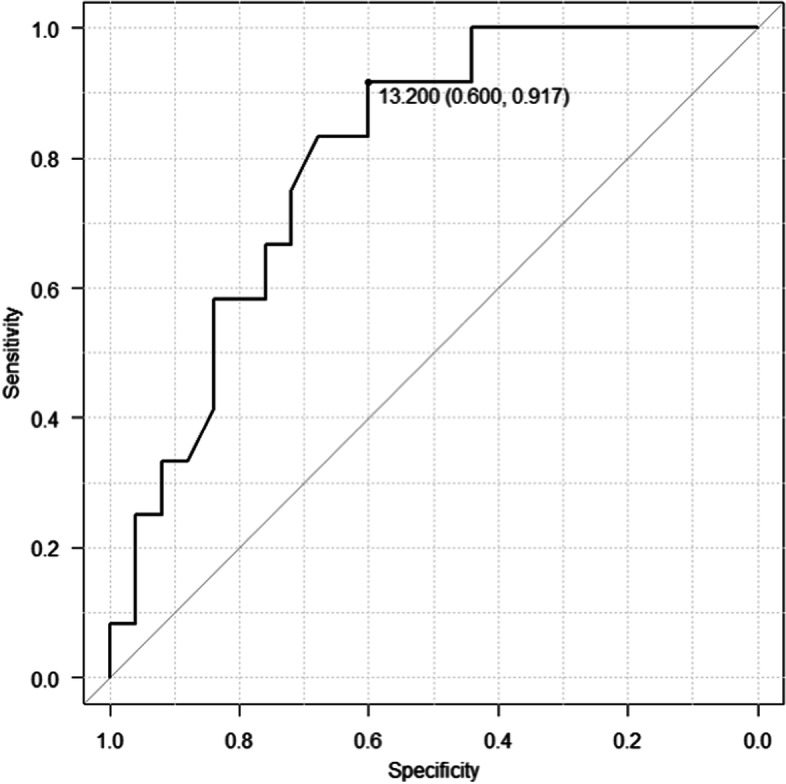
The receiver operating characteristic curve of the time from surgery to balloon dilation. The area under the curve was 0.80 (95% CI, 0.65–0.94) and the cut-off value was 13.2 months

Univariate and multivariate analyses using COX proportional hazards regression were performed to evaluate the possible risk factors for stricture recurrence after initial balloon dilation **(**Table 3). In the univariate analysis, time from surgery to balloon dilation (unit hazard ratio [HR] for month, 0.86; 95% CI, 0.76–0.98; *p*-value = 0.02) and residual waist during balloon dilation (HR, 6.67; 95% CI, 1.45–30.6; *p*-value = 0.01) were significant factor for CJS recurrence after balloon dilation. Multivariate analysis was performed for the above two factors, it indicated that both the time from surgery to balloon dilation (unit HR for month, 0.87; 95% CI, 0.76–0.99; *p*-value = 0.04) and residual waist during balloon dilation (HR, 5.46; 95% CI, 1.18–25.1, *p*-value = 0.03) were significant risk factors for the CJS recurrence after balloon dilation. The ROC analysis was performed for the time from surgery to balloon dilation, and the cut-off value was 13.2 months with area under the curve of 0.80 (95% CI, 0.65–0.94) (Fig. 4) The risk of recurrence was higher if the on-set of CJS is earlier than 13.2 months.

**Table 3:** COX proportional hazards regression for patency duration of anastomosis

	Univariate analysis		Multivariate analysis	
	Hazard ratio (95%CI)	*p* value	Hazard ratio (95%CI)	*p* value
Gender (male)	1.89(0.50–6.97)	0.34		
Age (year old)	1.01(0.95–1.07)	0.65		
modified Child's method reconstruction	3.11(0.40–24.1)	0.27		
Malignant disease	1.27(0.34–4.69)	0.72		
Time from surgery to initial BD (month)	0.86(0.76–0.98)	0.02	0.87(0.76–0.99)	0.04
Diameter of bile duct above CJS (mm)	0.81(0.63–1.04)	0.1		
Balloon diameter (mm)	0.77(0.51–1.14)	0.2		
Residual waist	6.67(1.45–30.6)	0.01	5.46(1.18–25.1)	0.03
Presence of bile duct stone	1.19(0.38–3.74)	0.78		
Presence of scar like appearance	1.35(0.43–4.26)	0.61

## Discussion

In our study, the technical success rate, clinical success, and adverse event rates were 92%, 100%, and 0%, respectively. The reasons for technical failure were not failure of balloon dilation itself, but scope insertion and anastomosis site detection. CJS recurrence after balloon dilation was observed in 12 patients, with a median follow-up period of 11.5 months (range, 0.8–97.5). Multivariate analysis showed that the time from surgery to balloon dilation and residual waist during balloon dilation were significant risk factors for CJS recurrence.

The development of BE has led to the creation of a new technique called BE-ERCP, which is considered a minimally invasive treatment for CJS. Although no standard treatment has been established for CJS, the Asia–Pacific Consensus Guidelines for the endoscopic management of benign biliary strictures [6] recommends dilatation with a balloon catheter as the first-line treatment, and balloon dilation is the generally used treatment for CJS in actual clinical practice. A study by Mizukawa et al*.* [9] retrospectively evaluated balloon dilation for CJS during BE-ERCP in patients with a prior Whipple procedure, and reported 100% of success rate (46/46). In contrast, Sakakihara et al*.* [10] retrospectively evaluated the management of CJS using BE-ERCP in 44 CJS patients (27 modified Child’s method reconstruction, 17 Roux-en-Y [R-Y] reconstruction), and reported that technical failure occurred in 6 patients with R-Y reconstruction because of difficulty of scope insertion. A retrospective study by Tomoda et al*.* [11] similarly reported that, scope insertion failures occurred only in 1 out of 113 patients (0.9%) with modified Child’s method reconstruction, whereas the scope insertion failed in 10 out of 63 patients(15.9%) with R-Y reconstruction. In our study, two of the three patients of technical failure were scope insertion failures. One of these two patients was an R-Y reconstruction, and the other was a special PD reconstruction (Osada method, which has a long afferent limb compared to Child’s method). The reported technical success rates of balloon dilation for the CJS are high, but scope insertion into the CJS remains challenging in patients with a long afferent limb, such as R-Y reconstruction. Conversely, both Sakakihara et al*.* [10] and Tomoda et al*.* [11] reported that the technical success rates for balloon dilation of CJS were very high in patients who had successful scope insertion, and were 95% (36/38) and 96% (133/139), respectively. In addition, in this study as well, the technical success rate in patients with successful scope insertion was 97% (37/38), and that of balloon dilation for CJS was attributed to the scope insertion into CJS. The clinical success rates of balloon dilation for CJS have also been reported to be extremely high, with the studies by Mizukawa et al*.* [9] and Tomoda et al*.* [11] reporting 100% of clinical success rates (46/46 and 103/103, respectively) in the patients who achieved technical success. In our study, the clinical success rate was 100% (37/37), once the technical success was achieved. Favorable short clinical outcomes can be expected if technical success is achieved in balloon dilation of the CJS during BE-ERCP.

A certain ratio of CJS recurrence has been reported in the long-term outcomes of balloon dilation for CJS. A retrospective study by Mizukawa et al*.* [9] reported that cumulative patency rates at 1, 2, and 3 years after balloon dilation were 73%, 55%, and 49%, respectively, in 42 patients who underwent balloon dilation for CJS, and was 57% (24/42) of total CJS recurrence rate with a median follow-up period of 3.5 years (range, 1.9–5.0 years). Another retrospective study by Tomoda et al*.* [11] reported the recurrence rates of 37.5% and 53.4% at 1 and 3 years, respectively, after balloon dilation in 103 CJS patients. Sano et al*.* [12] retrospectively evaluated long-term follow-up outcomes of 61 patients who underwent balloon dilatation for CJS (11 patients with stents), and reported that the CJS recurrence rate was 31% (19/61), which was a relatively high. In this study, CJS recurrence was observed in 32% (12/37) of patients who underwent balloon dilation for CJS with a median follow-up period of 11.5 months (range, 0.8–97.5) after the initial balloon dilation. According to the results of the above studies, including the current study, balloon dilation alone is insufficient for the management of CJS in a certain proportion of patients, even with favorable short-term outcomes.

Regarding the risk factors for recurrence of CJS after balloon dilation, time from surgery to balloon dilation (unit HR for month, 0.87; 95% CI, 0.76–0.99; *p*-value = 0.04), and residual waist during balloon dilation (HR, 5.46; 95% CI, 1.18–25.1; *p*-value = 0.03) were significant risk factors of CJS recurrence in our study. The ROC analysis of the time from surgery to balloon dilation showed that the cutoff value was 13.2 months (area under curve, 0.8; 95% CI, 0.65–0.94), which means the risk of CJS recurrence was higher if the on-set was earlier than 13.2 months. A few studies have evaluated risk factors for recurrence. Tomoda et al*.* [11] and Sato et al*.* [8] reported that, a time from surgery to balloon dilation of less than 1 year, was associated with a significantly higher rate of CJS recurrence in patients treated with balloon dilation alone. Sato et al*.* [8] also found that the absence of a scar-like appearance around the anastomosis was a significant risk factor for CJS recurrence. Sano et al*.* [12] reported significantly higher CJS recurrence in patients with a residual waist during balloon dilation. Although the residual waist during balloon dilation could be risk factor for CJS recurrence, the balloon diameter used for dilation was not a significant risk factor for CJS recurrence in this study same as previous report [8, 11, 12]. In our study, in the 18 patients with the residual waist at balloon dilation, the balloon diameters used were 6 mm in 3 patients, 8 mm in 11 patients, 10 mm in 1 patient, and 11 mm in 3 patients, in the 19 patients with no residual waist were 6 mm in 2 patients, 8 mm in 10 patients, 10 mm in 5 patients, and 12 mm in 2 patients. There was no significant difference in balloon diameter between the two groups (*p*-value = 0.1). This suggests that there might be no relationship between balloon diameter and residual waist, although there is a possibility of lack of statistical power because of the small cohort size. Since there have been few reports regarding risk factors for CJS recurrence, further studies are required, to identify patients with the known risk factors.

Further efforts to obtain more favorable long-term outcomes after balloon dilation for CJS, and stent placement after balloon dilation may be a good option, especially in patients with the known risk factors for CJS recurrence after balloon dilation. Stent placement is recommended in the Asia–Pacific consensus guidelines for the endoscopic management of benign biliary strictures in patients with recurrent biliary strictures after balloon dilation [6], although the recommendations are considered for patients with normal anatomy. Tomoda et al*.* [11] retrospectively compared 29 CJS patients treated with plastic stenting and 103 patients treated with balloon dilation. The results showed that the 1-year recurrence-free rates were 62.5% in the balloon dilation group, and 89.4% in the plastic stenting groups, with significantly fewer recurrences in the plastic stenting group. In addition, Tomoda et al*.* [13] prospectively studied 40 patients who underwent plastic stenting for CJS with only 3 (8.3%) CJS recurrences in a median follow-up of 21.3 months (range, 15.2–39.7 months), and showed long-term efficacy of plastic stenting. Long-term benefits have also been reported. Sato et al*.* [14] also reported the treatment of fully covered metallic stent deployment in 20 CJS patients, wherein only one patient (5.9%) had CJS recurrence after a median follow-up duration of 11.9 months. In our study, 8 of the 12 patients with CJS recurrence were successfully treated with fully covered metallic stent deployment, and no procedure-related complications were observed. No CJS recurrence occurred after stent removal (a median follow-up period of 13.3 months range, 7.0–30.8), so the long-term results of additional stent placement are considered as favorable as previously reported [14]. Stenting after balloon dilation may be an effective treatment option in patients with a higher risk of CJS recurrence, although another endoscopic session is required to remove the stent, which might be challenging because of the altered anatomy. Regarding adverse events caused by metallic stent deployment, there is a report of a patient who underwent small intestinal ileus due to migration of the metallic stent [15]; hence, careful follow-up after metallic stent deployment is necessary. Additionally, because of the scarce evidence regarding stenting for CJS, further evaluations regarding the optimal stent selection and placement duration, as well as its efficacy and safety, are warranted.

Recently, the usefulness of endoscopic ultrasound (EUS)-guided treatment for CJS patients with a surgically altered anatomy has also been reported. Although BE-ERCP is a useful procedure with a high success rate, it is not suitable for patients for whom it is difficult to insert the scope or identify the anastomotic site. For the treatment of such patients, EUS-guided biliary drainage (EUS-BD) may be a good indication. EUS-BD allows access to the bile duct without the need to reach the anastomosis site with an endoscope. This is done by puncturing the intrahepatic bile duct in the left lobe of the liver from the residual stomach or the anastomosed small intestine under EUS guidance. In the treatment of bile duct stones, EUS-guided treatment is reported to have a relatively high success rate of 60–100% [16]; therefore, it is an effective treatment. Iwashita et al*.* [17] retrospectively evaluated the outcomes of 23 patients with EUS-guided antegrade treatment, and 96 patients treated with BE-ERCP for bile duct stones. They reported that the treatment success rate was 65.2% (15/23) vs. 69.8% (67/96), and the adverse event rates were 17.4% (4/23) and 7.3% (7/96), respectively, with no significant difference, and comparable treatment outcomes between the methods. EUS-guided antegrade balloon dilation may be useful for patients who have difficulty with scope insertion [18], and the EUS-guided rendezvous technique [19] may be effective for patients who have difficulty in finding the anastomosis site. Because EUS-guided treatment has also been reported to have outcomes comparable to those of BE-ERCP, it may be useful as a salvage treatment for unsuccessful BE-ERCP cases. In fact, there were 2 patients that EUS-BD was performed as initial treatment for CJS, but they were excluded from this study because CJS patients with BE-ERCP as initial treatment was an inclusion criterion. Both of these patients were referred to our department for EUS-BD due to failure of BE-ERCP, they were successfully treated by EUS-BD. In addition, there have been some reports of other techniques for difficult cases. Fujita et al*.* [20] recognized bile leakage from strictures by filling the intestinal lumen with water and found the anastomosis. Mandai et al*.* [21] reported a case in which an ultraslim endoscope was inserted directly into the bile duct via the EUS-BD route, and the patient was treated with CJS under direct vision. These techniques may be useful in cases in which an anastomosis cannot be found. Murakami et al*.* [22] successfully treated a patient with severe colon anastomosis stricture in postoperative colon cancer by using an injection needle catheter, puncturing and injecting contrast medium to confirm the lumen, and then cutting the stricture with a needle knife, which may also be applicable to CJS. However, there are only few reports, and further studies are required for such difficult cases.

This study had several limitations. Due to the retrospective study design with a small cohort, a selection bias may exist. In addition, the external validity may be low because this study was conducted at only two centers.

## Conclusion

Balloon dilation for the CJS using BE-ERCP has shown favorable technical and short-term clinical success rates. However, CJS recurrence was observed in approximately one-third of patients after successful balloon dilation. Earlier occurrence of CJS, especially within less than 13.2 months, and residual waist during balloon dilation were considered risk factors for recurrence. Further studies on the long-term outcomes of CJS management and stenting, or other treatments in patients with risk factors of CJS recurrence are warranted.

## Data Availability

The datasets used and/or analysed during the current study are available from the corresponding author on reasonable request.

## Abbreviations

CJS: Choledochojejunal anastomotic stricture
PD: Pancreaticoduodenectomy
BE-ERCP: Balloon endoscopy-assisted endoscopic retrograde cholangiopancreatography
PTBD: Percutaneous transhepatic biliary drainage
DBE: Double balloon endoscopy
CI: Confidence intervals
ROC: Receiver characteristics operating curve
HR: Hazard ratio
R-Y: Roux-en-Y
EUS: Endoscopic ultrasound
EUS-BD: EUS-guided biliary drainage
